# Artificial Neural Networks for Modeling Mechanical and Microstructural Properties of Spark Plasma Sintered Powders

**DOI:** 10.3390/ma19050848

**Published:** 2026-02-25

**Authors:** Katarzyna Peta, Jakub Wiśniewski, Piotr Siwak

**Affiliations:** 1 Institute of Mechanical Technology, Poznań University of Technology, 1 Rychlewskiego, 61-131 Poznań, Poland; 2 Łukasiewicz Research Network—Poznań Institute of Technology, 6 Ewarysta Estkowskiego St., 61-755 Poznań, Poland; jakub.wisniewski@pit.lukasiewicz.gov.pl

**Keywords:** spark plasma sintering, artificial neural networks, mechanical properties, microstructure, functional surfaces, industrial applications

## Abstract

This paper presents a novel approach to modeling and optimizing the mechanical and microstructural properties of 316L stainless steel surfaces manufactured by spark plasma sintering (SPS). The integration of artificial intelligence techniques, particularly artificial neural networks (ANNs), with the optimization of material properties and the spark plasma sintering (SPS) process reflects the growing emphasis on intelligent manufacturing in advanced industrial applications. The surface functionality depends on the material’s mechanical and microstructural characteristics. The optimization technique was developed through the processing of a comprehensive set of measurement data, forming the foundation for the artificial intelligence method. To model the relationships between SPS parameters (sintering temperature and holding time) and material properties (density, porosity, hardness, and surface-affected zone (AKA the possible carbide zone depth), a series of controlled experiments was conducted. The performance of neural network models was evaluated using their coefficients of determination (R^2^ > 0.95) and the sum of squared errors (SSE < 0.02). These metrics were calculated by comparing actual measurement data with values predicted by the models. Validation experiments confirmed the reliability of the presented models and their relevance for implementation in industrial environments. The predictive model is valid for 316L stainless steel within the tested SPS setup and parameter range.

## 1. Introduction

Spark plasma sintering (SPS) is one of the most advanced methods for densifying a broad spectrum of powder materials from many material groups, including metals, ceramics and composites [1,2,3,4]. The main advantages of SPS are short processing times, the possibility of obtaining very high heating rates (around 1000 °C/min), reduced sintering temperature, limited grain growth, and the possibility of sintering various materials, including those difficult to sinter (such as refractory metals and their carbides) [5,6,7,8]. The working principles of this method are based on the simultaneous pressing and heating of the powder material. The heating is realized through Joule’s heat generation and the presence of electrical discharge between powder particles [9,10]. Those mechanisms are possible due to the pulsed direct current flow through the sintered material (whenever it is electrically conductive) and the sintering tools, which are usually made of graphite [11]. The usage of graphite tools can lead to microstructural changes which are, in most cases, undesirable. Due to the carbon-rich conditions in sintering tools, thermally driven diffusion of carbon to the sintered material is a common phenomenon mentioned in a significant number of papers [12,13].

Materials sintered by the SPS method are primarily characterized by density and porosity, which are used to analyze the densification process. In the case of most engineering materials, mechanical properties such as hardness, fracture toughness and tensile or compressive strength are also often determined. The surface properties of a material can also influence a number of functional properties, such as the wettability [14], corrosion [15], and fatigue strength [16]. While the main goal of the sintering process is the densification of the material in powder form, some of the materials are manufactured via a reactive sintering process [17]. In such cases, the formation of new phases, changes in the existing phases’ stoichiometries, and the homogenization of chemical and/or phase composition are, along with densification, equally important [18]. Regarding the usage of graphite sintering tools, changes in the material’s microstructure can be caused by the thermally driven diffusion of carbon from the tool to the sintered material [19]. While sintering metals with high carbide formation potential, this process can significantly change the properties of the sintered material, especially near its surface. Diffusion of the carbon can be limited by the usage of thin tungsten foil placed around the sintered powder; however, such a solution is economically reasonable only in some cases due to the high cost of tungsten foil [20]. Thus, the possibility of predicting the depth of microstructural changes in the material due to carbon diffusion could be crucial in optimizing the sintering process, particularly regarding the indication of process parameter thresholds for the determination of the necessity of usage of tungsten foil [21]. The mechanical and microstructural properties of powder metal sinters affect functionality, e.g., the mechanical strength and corrosion resistance [22]. SPS parameters can not only affect the internal structure of materials but also aspects of the surface topography. Therefore, surface phenomena, e.g., wettability [14,23], corrosion [24] and friction wear [25], can be indirectly determined.

Artificial neural networks can be fundamentally useful in modeling the relationships between manufacturing process parameters and material [26], mechanical [27], and functional properties [28]. The benefit of ANNs is solving problems associated with optimizing the properties of metal powder sinters, which are difficult to describe with classical mathematical models. This difficulty results from the complexity of the interactions between the SPS process and the properties of sinters [29]. However, ANNs are not “black boxes”, and it is possible to generate mathematical models [30]. ANNs make prediction easier than with other classical mathematical modeling methods, and they are based on creating a reliable knowledge base built on comprehensive measurement results and formulating relationships between input and output factors [31]. Due to the complex relationships among process parameters, deep learning combined with optimization methods, including Bayesian approaches, provides an effective way to identify optimal settings [32]. Deep learning-based methods, complementing existing physical models, have shown promise for manufacturing process control [33].

The integration of artificial neural networks in SPS research demonstrates diverse computational approaches for material property prediction and process optimization. Adesina et al. studied a back-propagation artificial neural network (ANN) with a 2-12-2 architecture and Levenberg–Marquardt algorithm for GNP/PLA (graphene nanoplatelet/polylactic acid) nanocomposite sintering. Input parameters included the sintering temperature and pressure, while outputs focused on density and hardness. Experimental data from SPS-processed samples with varying GNP content were used for training, achieving correlation coefficients of *R* = 0.955 for density and *R* = 0.983 for hardness [34]. A similar approach (to predict the properties of the materials obtained with the different process parameters) was described by Chakravarty et al. [35]. The study focused on the mechanical properties of zirconia-toughened alumina (ZTA) obtained via SPS sintering. An analysis of the influence of the chemical composition (zirconia addition), sintering temperature and heating rate on the hardness and fracture toughness of the material was performed. An ANN model describing the relationship between the sintering process parameters and the mechanical properties of ZTA was developed. In addition, the importance of each process parameter regarding the material’s properties was determined—the percentage of zirconia and the sintering temperature were identified as the most crucial factors. ANNs were also used by Mohammadzadeh and Aghaeinejad-Meybodito [36] to model and predict the effects of Ni content and sintering temperature on the densification and microhardness of WC-Ni nanocomposites processed via SPS. A feed-forward backpropagation ANN (2:5:2 topology, trainlm algorithm) showed good agreement with experimental data (R^2^ = 0.9983 for density and R^2^ = 0.9924 for hardness), accurately capturing the nonlinear sintering behavior. The model revealed that the sintering temperature had a stronger influence (62%) than the Ni content (38%) with optimal properties achieved at 12 wt% Ni and 1460 °C. Another study focusing on the optimization of composite material properties was presented by Maurya et al. [37]. Properties such as the effective density, relative density and hardness were analyzed as functions of the process parameters (sintering temperature and holding time) as well as functions of composition. In the referred work, the researchers studied titanium alloy (Ti6Al4V) reinforced by nano-sized TiN particles. The reportedly high accuracy of the proposed ANN model emphasizes the potential of neural networks in materials optimization. The genetic algorithm (GA) and particle swarm optimization (PSO) were used with an integrated ANN by Velmurugan and Senthilkumar [38] to perform SPS process optimization. The study shows the applicability of ANNs in the manufacturing of metallic materials such as NiTiCu shape memory alloy. The results showed that enhancements in the material density and hardness can be achieved by the reduction in the particle size and the increase in the sintering pressure and temperature. Research presented in [39] shows the possibility of using ANNs in unconventional SPS processes, such as joining two materials. The ANN model with 4-9-9-1 architecture was constructed to investigate the relationship between bonding parameters and the shear strength of the TZM–graphite joint. The correlation coefficient of r = 0.99614 confirmed the accuracy of the proposed model despite the complexity and nonlinearity of the dependencies. Jajarmi et al. presented an adaptive neuro-fuzzy model predicting the density and hardness of the spark plasma sintered materials. The determination coefficient R^2^ for the prediction of sintering properties is 0.9689 [40].

Although ANNs have been used to analyze the influence of the spark plasma sintering parameters on the densification of materials and their mechanical properties, researchers have not yet considered a comprehensive prediction of both the mechanical properties and the microstructure of 316L stainless steel. The use of ANNs to predict the microstructures of materials obtained by SPS sintering has not been studied in depth, and thus the reliability of this method in the context of predicting the microstructural aspects of materials such as the surface-affected zone (possible carbide formation) has not been thoroughly investigated. This paper presents the results of measurements of the mechanical and microstructural properties of spark plasma sintered materials resulting from specific SPS parameters. On this basis, an artificial neural network model was developed to predict these relationships. The most important parameters of the ANN model influencing the effectiveness of the neural network were also identified.

This paper aims to present a novel approach for selecting optimal spark plasma sintering (SPS) parameters in order to achieve the expected mechanical and microstructural properties of metal powder sinters. The reverse process is also included, involving the prediction of sintering properties based on the selected process parameters. Therefore, this bidirectional prediction approach aims to improve the applicability of measurement systems. These studies contribute to the advancement of measurement prediction approaches aimed at accurately quantifying essential sinter characteristics such as density, porosity, hardness, and the surface-affected zone (also referred to as the possible carbide zone depth) under various spark plasma sintering conditions. An assessment of the artificial neural network models based on experimental data confirms the reliability and practical value of integrating actual measurement data with values predicted by the models. These studies have a wide range of industrial applications, as advanced materials produced by spark plasma sintering are characterized by a fine microstructure, high density and the expected, enhanced functional properties [41]. These features are desirable in, among others, cutting inserts, implants, armor plates, or magnetic actuators [42]. The predictive model is reliable within the tested SPS setup and sintering parameters. In these studies, 316L stainless steel is investigated. Therefore, potential applications are focused on surgical instruments, medical implants, tool holders, precision mechanical parts (bushes, guides, sealing rings), anti-corrosion parts of the chemical and food industry (nozzles, valves), filters, and elements of measuring instruments [43].

## 2. Materials and Methods

### 2.1. Material

In these studies, the powder of 316L (1.4404) stainless steel (APS 15–45 µm, Renishaw, New Mills, UK) was used. The chemical composition of 316L is given in Table 1.

### 2.2. Spark Plasma Sintering

The consolidation of powders was performed by the SPS method using HP D 25/3 (FCT System, Frankenblick, Germany). Weighted powders were placed in sintering tools made of fine-grained 2334 graphite grade (Mersen, Courbevoie, France) and then sintered under vacuum (0.05 mbar) with the following stable parameters: compaction pressure of 50 MPa, heating rate of 100 °C/min and cooling in a furnace. Input parameters for model training—sintering temperature and holding time—varied from 950 °C to 1150 °C and from 1 to 15 min, respectively. Validation of the model was performed based on materials obtained at 975 °C and 1075 °C held for 12 and 3 min, respectively. All samples were manufactured with the same size of 20 mm in diameter and 5 mm in height. The selection of SPS parameters for training the artificial neural network was dictated by the previous experience of qualitatively acceptable sinters [44]. The neural network was constrained by these limit values so as not to produce defective sinters. The defects of metal powder sinters mainly include excessive porosity, insufficient powder grain fusion, partial melting, and excessive grain growth. Incorrectly selected SPS parameters can lead to sinter deformation, or lower mechanical strength, resulting in cracks [45].

### 2.3. Density Measurements

After sintering, all samples were sandblasted with glass micro-balls to remove residual graphite foil used in the sintering tool assembly process. The process was carried out with the lowest possible pressure to prevent any material loss from the sample surfaces. After cleaning the samples, the density of materials was measured following the Archimedes principle and the ISO 18754:2020 [46] standard using an Explorer EX225DM (OHAUS, Nänikon, Switzerland) laboratory scale. Each density measurement was repeated three times and averaged.

### 2.4. Porosity Measurements

In this paper, two different measures of porosity are used. Open porosity quantifies the fraction of interconnected pore volume using the Archimedes method, providing a volumetric perspective, while microscopy-based porosity measures the proportion of voids in a 2D image, offering a planar perspective.

Measurements of density using the Archimedes principle provided data to calculate the open porosity of the materials. Open porosity was determined using the Archimedes method based on fluid immersion. Prior to the measurements, the samples were dried to a constant mass and weighed in air to obtain the dry mass. The specimens were then fully immersed in the fluid and kept under immersion until complete saturation. Then, the samples were weighed in air to determine the saturated mass and subsequently weighed while immersed in the fluid to obtain the apparent immersed mass. The open porosity was calculated from the resulting mass differences, which allow for the quantification of the volume of fluid within the interconnected pores.

**Figure 1:**
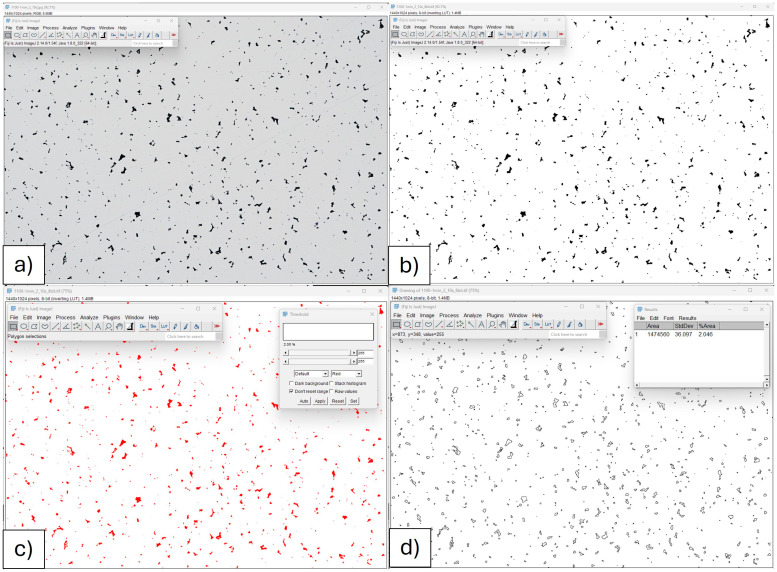
Methodology of image processing for porosity measurements: (**a**) original microphotograph, (**b**) brightness and contrast adjustment with image binarization, (**c**) application of the mask according to the color threshold, (**d**) outlining of the pores with the percentage content of the selected area—pores. Note: The sample microphotograph contains a porosity of 2.046%.

In addition, the overall porosity (including both closed and open pores) was calculated based on the microstructure observations. All samples were cut in half and mounted in resin. Then, grinding and polishing were performed to provide a mirror-like surface of the samples. Observations of the non-etched microstructure were taken using an Eclipse L150 optical microscope (Nikon, Tokyo, Japan) equipped with NIS Elements image analysis software (https://www.microscope.healthcare.nikon.com/en_AOM/products/software/nis-elements, accessed on 10 February 2025). All non-etched images were collected with enhanced contrast to enable accurate porosity measurements. Then, image analysis was performed using ImageJ software, version 1.54f (National Institutes of Health, Bethesda, MD, USA). Image preparation included binarization of the images, noise cancellation, and the measurements of porosity such as the percentage of the black area in the image. Porosity derived from image analysis was quantified as a two-dimensional area fraction, which was calculated as the ratio of the total pore area to the total analyzed image area. As this approach is based on two-dimensional cross-sectional images, the resulting value represents an area fraction rather than a true volumetric porosity. The accuracy of this method depends on factors such as the representativeness of the analyzed sections. Therefore, all values of porosity were calculated based on three areas from each sample. The representative porosity analysis is presented in Figure 1.

### 2.5. Microstructure Measurements—Surface-Affected Zone

**Figure 2:**
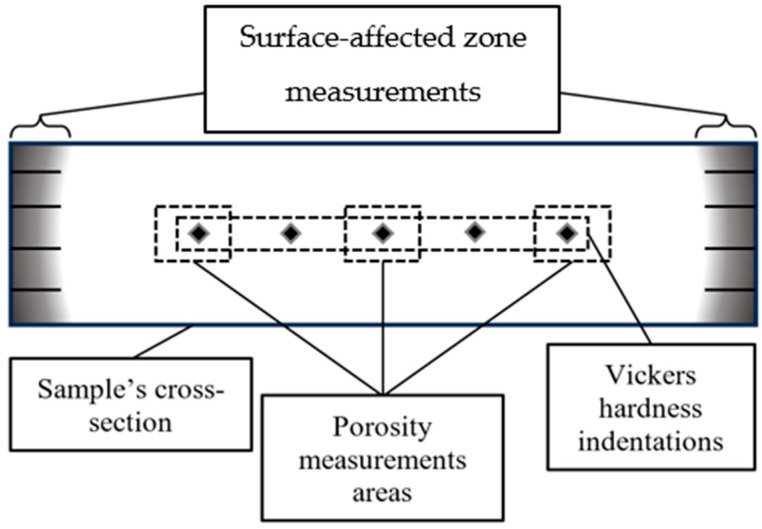
Schematic overview of the measurement areas on the cross-section of the sample.

After observations of the non-etched surface of the material, all samples were cleaned with ethanol and etched using a two-component glycerin–acid etching reagent for stainless steel (glyceregia). The surface-affected zone depth was determined from conventional optical micrographs of polished and etched cross-sections. The surface-affected zone is a near-surface zone that is clearly microstructurally different from the bulk, as shown in Figure 2. The surface-affected zone was defined as the near-surface region exhibiting a higher areal density of likely carbides relative to the bulk. The boundary of the surface-affected zone was identified as the position at which possible carbides were no longer continuously present, and their local distribution became indistinguishable from that of the core microstructure at the applied magnification. The surface-affected zone was measured as the perpendicular distance from the surface to this boundary. The microstructure observations were performed to measure and calculate the surface-affected zone from eight measurements (four measurements from the left and right sides of the sample; see Figure 2). In all cases, the microstructure affected by carbon diffusion was considered the “carbide zone”, as it contained carbides of Cr_x_C_y_ stoichiometry. Thus, the surface-affected zone (also referred to as the possible carbide zone) should be considered as the part of the material with a different microstructure than the core of the material.

An Aeris (PANalytical, Almelo, The Netherlands) diffractometer equipped with a Cu lamp (Kα radiation, 1.5406 Å wavelength) was used in the 2θ range of 30–90° with a step size of 0.0109°, a time per step of 37.995 s, a voltage of 40 kV, and a current of 15 mA. The diffractograms obtained were analyzed qualitatively using HighScore Plus version 5.1a software and the PDF-4+ICDD database.

### 2.6. Hardness Measurements

Vickers hardness was calculated from measurements performed under a load of 2.942 N using an FM-800 (Future-Tech, Kanagawa, Japan) hardness tester, following the ISO 6507-1:2023 standard [47]. The hardness of each sample was expressed as the average value of five measurements made along the centerline of the sample.

### 2.7. Scheme of the Measurements

A detailed scheme of the measurement areas of the surface-affected zone, Vickers hardness, and porosity is presented in Figure 2.

### 2.8. Artificial Neural Networks

**Figure 3:**
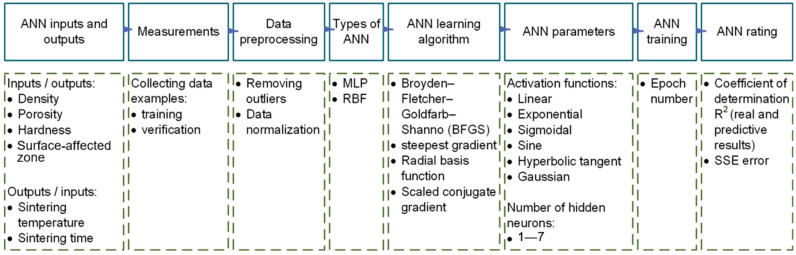
ANN modeling steps.

Modeling artificial neural networks requires considering the following steps: selecting network inputs and outputs, performing measurements to collect a set of ANN training and testing examples, preprocessing data, determining ANN types, selecting a training algorithm, determining ANN parameters, training the ANN, verifying the ANN, and assessing the ANN’s effectiveness (Figure 3). The characterization of the test data is presented in Section 3. The network’s performance was evaluated using test data that were not seen during training or validation, and the results are presented as graphs and statistical analyses in this paper.

After collecting the training data, preprocessing is recommended, including removing outliers [48] and normalizing the data [49]. Here, no outliers were noted. Data normalization is important to minimize the risk of inconsistency between groups of measurement data. In this study, the min–max function was applied, scaling all numerical data in various numerical ranges to the range from 0 to 1, according to Equation (1):

(1) $$x_{nor}=\frac{x-min(x)}{\max\left(x\right)-min(x)},$$

where *x_nor_*—normalized data value, *x*—data value directly from measurements, min(*x*)—minimum value in the measurement dataset, max(*x*)—maximum value in the measurement dataset.

The selection of the ANN architecture and parameter combinations was guided by the testing of 10,000 variants to cover the possible combinations of the permissible variables. The ANN architectures tested in this study included both multilayer perceptron (MLP) and radial basis function (RBF) networks. The number of neurons in the hidden layers was varied from 1 to 7. In addition, different learning algorithms were tested, including the Broyden–Fletcher–Goldfarb–Shanno (BFGS) algorithm, radial basis function (RBF), and scaled conjugate gradient. Moreover, various activation functions were also studied, such as linear, exponential, sigmoid, sine, hyperbolic tangent, and Gaussian. These combinations of network architectures and parameters were aimed at identifying the configurations that provide the most accurate predictions.

Two types of artificial neural networks were tested: multilayer perceptron (MLP) and the radial basis function network (RBF). The basic mathematical calculations in artificial neural networks are performed in neurons (network nodes), network layers (matrix multiplication) and in network-learning algorithms involving forward and backward propagation. The center of mathematical calculations is neurons. Here, input signals (*x_i_*) are multiplied by synaptic weights (*w_i_*) that weaken or amplify input signals, then a bias (*b*) is added, and the result of these calculations is activated by the activation function to finally generate an output signal (*y*) [50]:

(2) $$y=f(\sum_{i=1}^{n}w_{i}x_{i}+b)$$

Neural calculations (Equation (2)) are valid for the hidden and output layers of the MLP. However, in the RBF network, the output layer sums the results of calculations from hidden neurons containing radial basis functions. Here, the output signal (*y*) is the product of the synaptic weight (*w_i_*) and the radial basis function (∅) applied to the difference between the input (*x*) and central (*c_i_*) vectors, which is measured using the Euclidean distance (Equation (3)) [51]:

(3) $$y=\sum_{i=1}^{n}w_{i}\emptyset \left(\left\|x-c_{i}\right\|\right)$$

The most popular RBFs are Gaussian radial basis functions (Equation (4)) calculated from the width of the function (*σ*) and the Euclidean distance between the square of the input vectors and the center of the neuron. A function result close to 1 indicates the proximity of the input and center points, and therefore stronger activation, while a result close to 0 indicates the opposite [52].

(4) $$\emptyset \left(\left\|x-c_{i}\right\|\right)=e^{\left(-\frac{{\left\|x-c_{i}\right\|}^{2}}{2\sigma_{i}^{2}}\right)}$$

The learning algorithm is one of the determinants of the effectiveness of an artificial neural network. Here, the results of the Broyden–Fletcher–Goldfarb–Shanno, radial basis function, scaled conjugate gradient, and steepest gradient algorithms were tested. The BFGS algorithm is quasi-Newtonian. It is distinguished by modifying the synaptic weights based on the average gradient of the error after each epoch—a single pass through the training set. BFGS approaches the minimum of the error function using the Hessian—the matrix of second-order partial derivatives [53]. The BFGS method, which uses changes in synaptic weights in back-propagation [54], is considered to be one of the most effective quasi-Newton algorithms [55]. The radial basis function learning algorithm uses k-means clustering to find the centers for the RBF and calculate their spreads in order to later calculate the hidden layer outputs with a Gaussian function that considers the distances from the centers. The output layer outputs are generated using linear regression methods [56]. The scaled conjugate gradient algorithm cumulatively modifies the weights of synaptic connections after each epoch. The algorithm searches for a point with the minimum error value, creating conjugate directions with previously identified minima [57]. The steepest gradient algorithm minimizes the error function by modifying the synaptic weights toward a negative or positive gradient. This method is one of the first choices, because it is relatively fast and produces good results in simple networks. However, the effectiveness of this method is limited in multimodal tasks [58].

The ANN parameters chosen as modifiable are the activation functions (Table 2) and the number of hidden neurons. Therefore, during training and testing of the ANN, the influence of activation functions (linear, sigmoid, exponential, hyperbolic tangent, sine, Gaussian) and the number of hidden neurons (1–7) on the results was examined.

The process of training an artificial neural network is closely related to the concept of an epoch, which refers to a single pass through a prepared set of measurement results during training. Verifying the effectiveness of an ANN involves identifying the number of epochs needed to achieve a relatively low network error. Observing the number of epochs is important to minimize network errors (too few epochs) as well as the effect of overfitting (too many epochs) [60]. During training, the network minimizes a predefined error function, leading to a decrease in both training and validation errors. If the validation error ceases to decrease or begins to increase while the training error continues to drop, this indicates overfitting, in which the network becomes too closely fitted to the training set and loses generalization ability. Early stopping is applied by monitoring the validation error and stopping the training process when overfitting is detected, ensuring that the network maintains predictive performance on unseen data. This approach balances accurate fitting to the training data with robust generalization to new examples [61].

In the forward modeling approach used in this study, the artificial neural network predicts material properties, such as density, porosity, hardness, and surface-affected zone depth (likely carbide zone depth), from a given set of SPS process parameters, including temperature and sintering time, and it can, in principle, be used inversely to estimate process parameters from desired properties. In this context, the issue of non-uniqueness, where multiple parameter combinations could potentially lead to the same material properties, does not affect the network’s predictive performance in the forward direction. For any specific set of input parameters, the network produces a well-defined and consistent prediction of the corresponding properties. While the same properties could theoretically be obtained from different parameter combinations, the forward predictions remain valid and reliable for each individual parameter set. This approach is acceptable from an industrial perspective. However, it should be emphasized that the industrial applicability of the prediction model has been tested and found to be reliable within the scope of the tested SPS setup and sintering parameters.

The evaluation of the effectiveness of artificial neural networks was determined based on several basic criteria: the sum of squares error (SSE), mean squared error (MSE), root mean squared error (RMSE), and the linear coefficient of determination (R^2^) between the real results from measurements and the predictive results from the neural network [62]. A coefficient of determination closer to 1 indicates a better fit of both groups of data [63]. Linear regression analyses are often used because of their popularity and the ease of interpreting data by most scientists, resulting in more convenient comparisons of the data obtained in different studies [64]. The sum of squares error involves calculating the pooled variance [65], and it is popular for calculating the effectiveness of ANNs [66].

## 3. Results

### 3.1. Mechanical and Microstructural Properties

The density, open porosity, porosity, surface-affected zone and hardness of materials obtained in the SPS process with different sintering temperatures and holding times are given in Table 3. All presented values were used as a database for neural network testing and for comparison between the experimental results and the network predictions. Experimental data were selected from a technologically relevant range of SPS parameters (sintering temperature 950–1150 °C and sintering time 1–15 min) based on the authors’ experience and literature studies [67,68].

**Figure 4:**
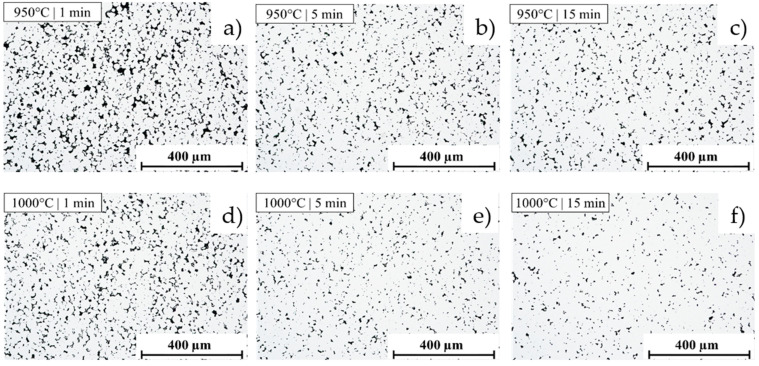

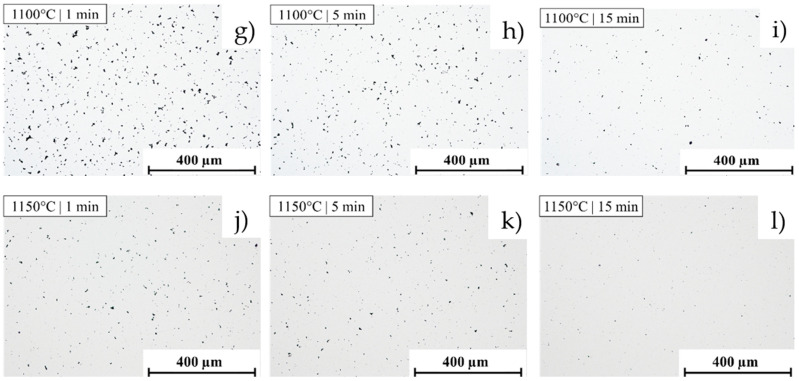
The microstructure of the materials obtained with the following sintering parameters (sintering temperature and holding time) is as follows: (**a**) 950 °C, 1 min; (**b**) 950 °C, 5 min; (**c**) 950 °C, 15 min; (**d**) 1000 °C, 1 min; (**e**) 1000 °C, 5 min; (**f**) 1000 °C, 15 min; (**g**) 1100 °C, 1 min; (**h**) 1100 °C, 5 min; (**i**) 1100 °C, 15 min; 1150 °C, (**j**) 1 min; (**k**) 1150 °C, 5 min; (**l**) 1150 °C, 15 min.

The microstructure of the materials obtained in the SPS process is presented in Figure 4, showing the densification dependence on both the sintering temperature and holding time.

**Figure 5:**
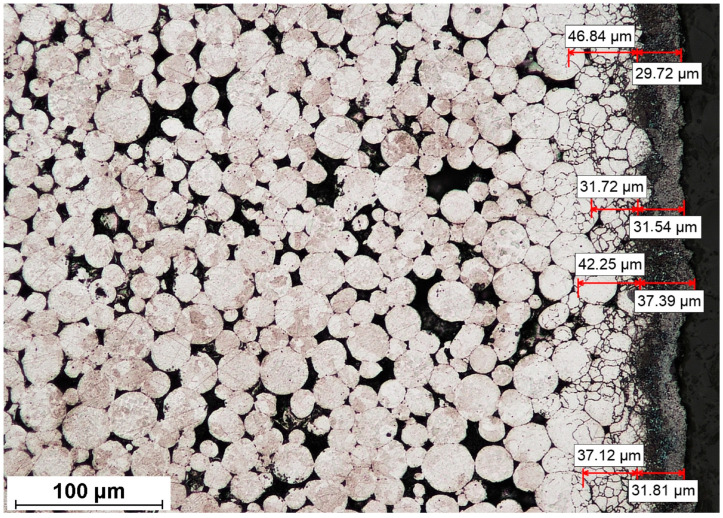
Representative surface-affected zone microstructure with the measurements of the two layers of the surface-affected zone.

**Figure 6:**
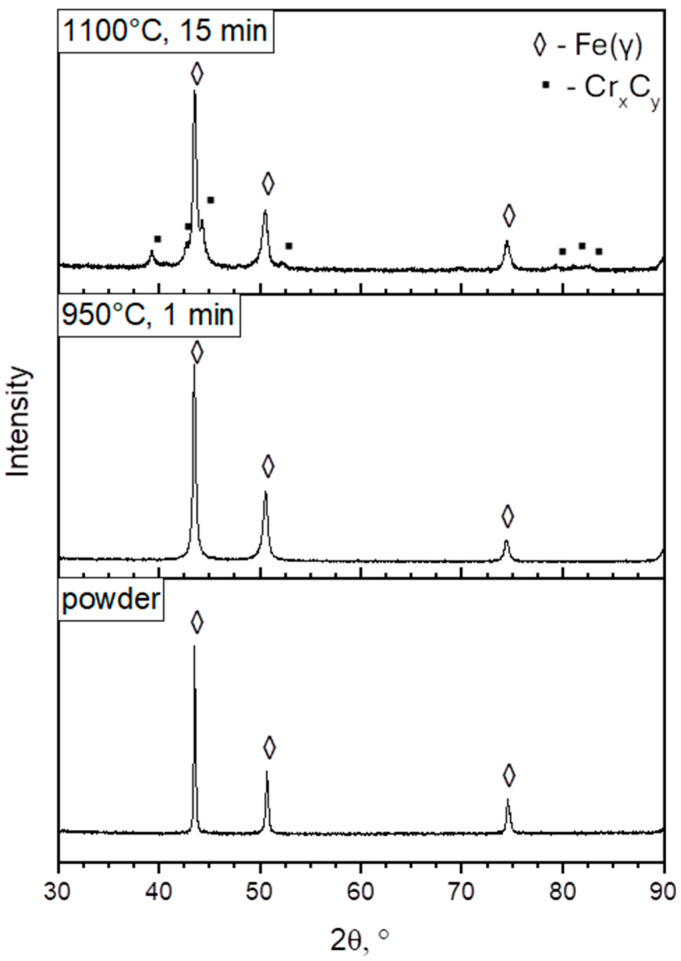
X-ray diffraction (XRD) patterns of 316L powder and sintered samples produced at 950 °C for 1 min and at 1100 °C for 15 min.

After the etching, the microstructure of the surface-affected zone was revealed (Figure 5), and the measurements of the surface-affected zone were performed. Observations of the etched samples demonstrated the layered character of the surface affected by the possible carbon diffusion. In addition, X-ray diffraction (XRD) patterns are presented in Figure 6.

The surface-affected zone (SAZ) was characterized to investigate the formation of possible carbide phases resulting from the spark plasma sintering (SPS) process of 316L stainless steel powders. An X-ray diffraction (XRD) analysis of selected samples sintered at 950 °C for 1 min and 1100 °C for 15 min revealed diffraction peaks that are consistent with Cr-rich carbides (Cr_x_C_y_). These results suggest that chromium carbides likely form in the SAZ due to thermal exposure during SPS.

### 3.2. Artificial Neural Networks

Two entirely independent modeling approaches were performed. In the first analysis, the dataset was divided into three subsets: a training set (80), a validation set (20), and a test set (14), which were each used exclusively within this modeling framework. This analysis was designed to evaluate various ANN architectures and parameter settings on a larger dataset with the aim of identifying the most effective model for generalization. In the second analysis, a leave-one-out cross-validation (LOOCV) procedure was conducted using only a 14-sample dataset. This analysis was intended to assess model stability and performance when training data are limited. For this purpose, a completely independent Python (version 3.13) implementation was developed, which operated exclusively on the same 14 samples previously designated as the test set in the first analysis. Importantly, this implementation did not use any model weights, parameters, or results from the first analysis, ensuring that the two approaches were entirely independent. As such, no data leakage occurred between the analyses.

In the first analysis, the most effective artificial neural network model was determined by evaluating the coefficient of determination, R^2^, and sum of squares error, SSE, across various ANN architectures and parameter settings, including the network type, the activation functions of hidden and output neurons, and the number of hidden neurons. An additional variable influencing the network efficiency was the number of epochs. In these studies, 10,000 variants of ANN models based on these parameters were generated.

The first prediction task was to estimate the mechanical and microstructural properties of 316L sinters (outputs) from the specified SPS parameters (inputs). Table 4 presents the most effective ANN models (R^2^ > 0.95) along with their model structure and evaluation criteria.

**Figure 7:**
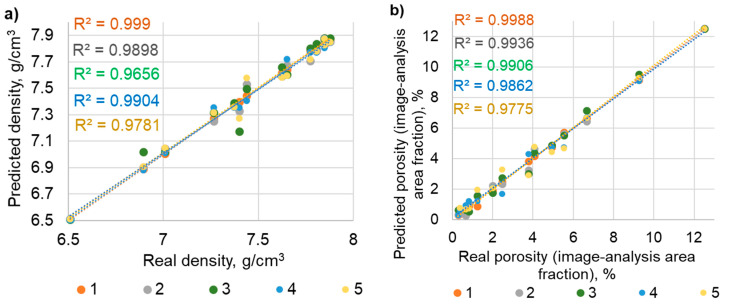

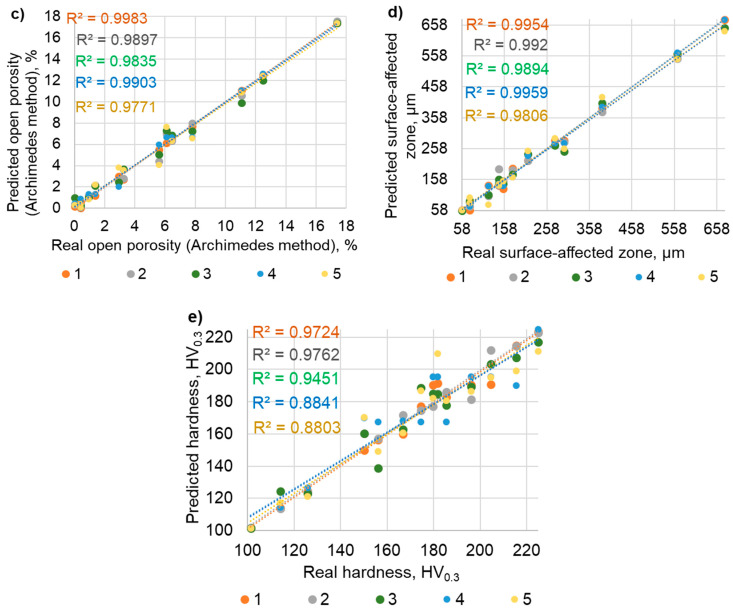
Linear regression of actual versus predicted values for SPS parameters (inputs) and sintering properties (outputs): (**a**) density, (**b**) porosity, (**c**) open porosity, (**d**) surface-affected zone, (**e**) hardness. Note: The legend on the graphs (1, 2, 3, 4, 5) refers to the ANN numbering in Table 4.

Figure 7 shows a linear regression graph between the real and predictive results. The fit of these two groups of data (real and predictive) is determined by the coefficient of determination R^2^. The regression plot compares specific real values and their corresponding predicted values. For better interpretation of the results, previously normalized data were reverse-normalized to the range from 0 to 1. Therefore, the values are read in the numerical measurement range as originally measured.

**Figure 8:**
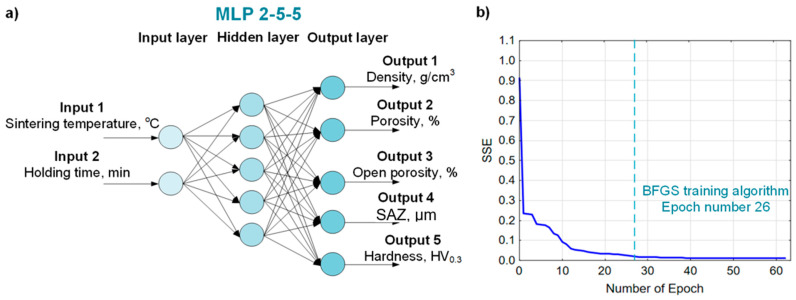
The most effective ANN for predicting sintering properties: (**a**) visualization of the neural network model architecture, (**b**) graph of the number of epochs versus the error (SSE).

The model of the most effective neural network for predicting the properties of sinters (density, porosity, hardness and surface-affected zone) based on spark plasma sintering parameters (sintering temperature, holding time) is shown in Figure 8a. Figure 8b presents a graph of the dependence of the sum of squares error (SSE) on the number of epochs. The optimal number of epochs for the prediction task (sintering properties) is marked, which allows the attainment of a relatively small error while avoiding overfitting. The most effective network is MLP 2-5-5 with an architecture of two input neurons, five hidden neurons, five output neurons, and the following parameters: network learning algorithm—BFGS, activation function of hidden neurons—hyperbolic tangent, activation function of output neurons—sigmoid. This neural network predicts all sintering properties with an average coefficient of determination (R^2^) value of 0.9967 and an SSE of 0.0015.

For the MLP 2-5-5 network, which was identified as the most effective for predicting the properties of sintered powders, the sum of squared errors (SSE), mean squared error (MSE), and root mean squared error (RMSE) were calculated, and both the real (measured) and predicted values are reported (Table 5). In this paper, predicted values were compared against real (measured) values, and performance metrics, including MAE and RMSE, were calculated on an unseen test set. Additionally, all normalization parameters were fitted exclusively on the training data and subsequently applied to the test set.

The second prediction task is to indicate spark plasma sintering parameters (output values) based on the mechanical and microstructural properties of the 316L sintered powders (input values). Providing the expected properties of sintered powders leads to the generation of recommended SPS process parameters by the ANN. Table 6 presents the most effective ANN models (R^2^ > 0.95) along with their model structure and evaluation criteria.

**Figure 9:**
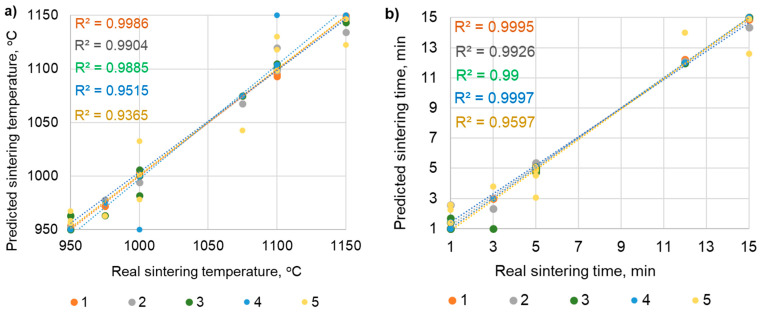
Linear regression graph comparing actual and predicted values for relationship between sinters’ properties (input) and SPS parameters (output): (**a**) sintering temperature, (**b**) holding time. Note: The legend on the graphs (1, 2, 3, 4, 5) refers to the ANN numbering in Table 5.

Figure 9 presents a linear regression graph comparing the real and predictive results along with the coefficient of determination R^2^. For the data presented in the graph, inverse normalization of the min–max function was applied.

**Figure 10:**
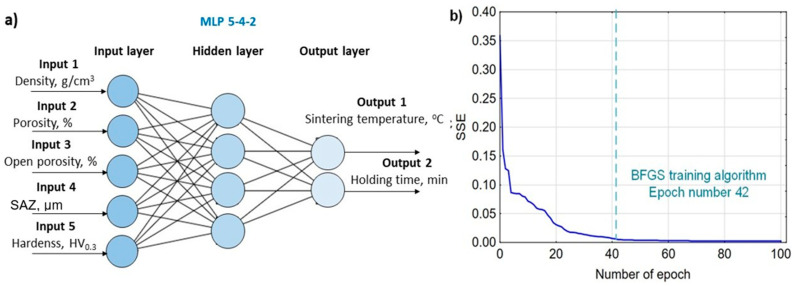
The most effective ANN for predicting SPS process parameters: (**a**) visualization of the neural network model architecture, (**b**) graph of the number of epochs versus the error (SSE).

The best model of the artificial neural network for predicting the SPS process parameters (sintering temperature and holding time) based on the sintered powders’ properties (density, porosity, hardness, and surface-affected zone depth) is shown in Figure 10. The most effective network is MLP 5-4-2 with an architecture of five input neurons, four hidden neurons, two output neurons, and the following parameters: network learning algorithm—RBFT, activation function of hidden neurons—exponential, activation function of output neurons—linear. This neural network predicts the SPS process parameters with an average coefficient of determination (R^2^) value of 0.99 and a sum of squares error (SSE) of 0.0001.

The multilayer perceptron (MLP) 5-4-2 network was identified as the most effective for predicting the sintering process parameters. The sum of squared errors (SSE), mean squared error (MSE), and root mean squared error (RMSE) were calculated to quantitatively assess its performance, and both the actual (measured) and predicted values are reported (Table 7).

In the second analysis, to report a robust validation strategy, a leave-one-out cross-validation (LOOCV) was applied to the testing dataset (14 observations). In each iteration, one sample was excluded from the dataset and used as an unseen test case, while the remaining 13 samples were used for training. This process was repeated 14 times so that each sample served exactly once as a test case. For every fold, input and output variables were normalized independently using min–max normalization to the range [0, 1], which was computed exclusively from the training dataset to prevent data leakage.

For validation of the model for predicting sintering properties, a multilayer perceptron (MLP) with a 2–5–5 architecture was employed consisting of two input neurons, one hidden layer with five neurons using the hyperbolic tangent activation function, and five output neurons with a linear non-negative activation function. Bias terms were included in both layers. Network weights were initialized randomly in the range [−0.1, 0.1] and optimized using the BFGS quasi-Newton algorithm to minimize the sum of squared errors (SSE) over the training data. To improve the stability of the training process, each fold was trained three times with different random initial weights, and the model yielding the lowest SSE on the test sample was retained.

For validation of the model for predicting spark plasma sintering parameters, a multilayer perceptron (MLP) with a 5–4–2 architecture was implemented. The network consisted of five input neurons representing the material properties, a single hidden layer with four neurons employing the hyperbolic tangent activation function, and two output neurons using a linear activation function. Bias terms were incorporated into both layers to enhance network flexibility. Initial weights were randomly assigned within the range [−0.1, 0.1] and subsequently optimized using the BFGS quasi-Newton method, minimizing the sum of squared errors between the predicted and real (measured) outputs. Prior to training, both inputs and outputs were scaled to the [0, 1] interval using min–max normalization, and predicted values were rescaled to their original units for evaluation. The model was trained under a leave-one-out cross-validation (LOOCV) scheme with each fold repeated three times using different random weight initializations. The iteration producing the lowest error on the test sample was selected.

After prediction, outputs were rescaled back to the original range. Model errors were evaluated using the mean absolute error (MAE) and root mean squared error (RMSE), which were computed for each output variable and averaged across all 14 folds. The models’ performance was quantified using errors calculated exclusively on unseen samples, ensuring that the results reflect true predictive outcomes. The leave-one-out cross-validation (LOOCV) results for predicting the sintering properties are presented in Table 8, while the prediction results for the spark plasma sintering parameters are shown in Table 9.

## 4. Discussion

These studies discuss the relationships between the manufacturing process and the mechanical and microstructural properties of 316L stainless steel, although the trends in the observed relationships can be applied to other spark plasma sintered materials. Spark plasma sintering parameters, namely the sintering temperature and holding time, are key to ensuring the durability of sintered metal powders. The properties of sinters that can indirectly influence long-term functionality are the porosity, hardness, and surface-affected zone depth.

Within the sintering temperature and holding time ranges recommended in these analyses (950–1150 °C, 1–15 min), their increase was found to improv the densification and hardness of the sinters while simultaneously reducing porosity. SPS parameters outside the recommended range can lead to poor powder particle bonding or excessive grain growth, resulting in reduced hardness. Electrical microdischarges between loosely spaced powder particles locally increase the temperature, thus promoting diffusion processes, and further compression extinguishes the electrical microdischarges, while the powder heats up mainly via resistance (Joule’s) heat. Therefore, SPS uses lower temperatures than other sintering processes by an average of up to 300 °C. This is a significant difference between the types of sintering processes, which is important for modeling and comparing experimental results.

The diffusion of carbon and the subsequent formation of carbides are well-known processes caused by the carbon-rich environment of graphite sintering tools. The use of tungsten foil or boron nitride (BN) spray can inhibit carbon diffusion; however, both solutions increase the cost and time required for tool setup and assembly [69]. Nevertheless, for materials that are very sensitive to carbon content, such as stainless steels, the precise control of carbon diffusion is crucial to achieve the desired properties of the material [70]. The main difficulties associated with carbide formation are increased surface hardness, which can significantly hinder the further machining of the material, and a reduction in, or even complete loss of, the corrosion resistance of stainless steel [71]. Thus, from a practical point of view, it is crucial to predict the depth of the material affected by microstructural changes, such as carbide precipitation. The accurate selection of sintering process parameters, supported by an ANN model, can be an effective way to obtain materials with a controlled surface-affected zone, without the need for costly modifications such as additional tungsten foil.

The spark plasma sintering parameters that primarily influence the mechanical and microstructural properties of sinters are the sintering temperature, sintering time, heating rate, pressing pressure, and atmosphere. Authors in the literature have undertaken studies to quantify the influence of individual parameters on the properties of sinters. Based on ANOVA (analysis of variance), it was identified that the most influential parameter is the sintering temperature, which is followed by sintering time as the second most influential parameter [72]. In other studies presenting a Pareto chart analysis for response, the sintering temperature is emphasized as the most important SPS parameter [73]. Thus, temperature is the key SPS parameter, which is followed by the time-related parameter, while the remaining parameters are also important for obtaining the desired sintering properties but to a lesser extent [74]. Therefore, the selection of SPS parameters for the neural network model was dictated by a review of studies in the literature.

In the spark plasma sintering process, the recommended range of parameters for manufacturing functional sintered 316L stainless steel is a sintering temperature of 950–1150 °C and a sintering time of 1–15 min [67,68]. An improper selection of sintering temperature and time in the SPS process can significantly influence the densification, microstructure, and mechanical properties of the material. Low sintering temperature or time can lead to high porosity, reduced relative density, and consequently lower mechanical strength. Conversely, excessively high temperature or prolonged sintering time can cause abnormal grain growth and the formation of secondary phases, which deteriorate the alloy’s mechanical properties. These extreme relationships have been discussed in the literature [75,76]; however, the aim of this paper is to study the parameters that are industrially and technologically relevant to achieve sinters of acceptable quality.

These studies focus on producing high-quality, industrially relevant sinters to ensure the practical applicability of the ANN models. Temperatures and holding times outside the tested range are considered out-of-spec conditions, requiring parameter adjustment. The goal is to ensure that the sinters achieve the best possible quality under realistic processing conditions. Deviations from the optimal SPS parameter range can significantly affect the microstructure and properties of the sinters. Excessive temperatures may induce abnormal grain growth and a coarsening of possible carbide phases, reducing hardness and mechanical strength [77]. Prolonged holding times can enhance diffusion and grain coalescence, potentially leading to microcracking or porosity development due to internal stresses [78]. Conversely, insufficient temperatures or short sintering times can result in incomplete densification, weak interparticle bonding, and increased porosity, limiting the mechanical performance and homogeneity of the sintered material [79]. Maintaining SPS parameters within the defined range is therefore critical to achieving sinters with consistent microstructural characteristics and industrially relevant mechanical properties.

The sintering temperature and holding time also influence the microstructural characteristics, grain size and surface-affected zone. In the SPS method, grain growth is significantly smaller than in conventional sintering methods due to the lower process temperature and shorter process time. However, an interesting microstructural aspect is the formation of the surface-affected zone which grows with increasing sintering temperature and holding time. Although possible carbide precipitation in the near-surface zone can be desirable in some applications, in the context of 316L steel, the presence of a surface-affected zone is highly undesirable. The formation of chromium carbides leads to decreased chromium content in the austenite, causing a loss of its anticorrosive performance. Similar results regarding the carbide zone depth to those obtained in the present study were reported by Pinot et al. The authors sintered 316L stainless steel using the SPS method, and the sample surfaces were analyzed. The formation of a carbide-rich zone near the surface was demonstrated, resulting from carbon diffusion from the tooling (graphite foil) into the sintered material [80].

The selection of optimal process parameters for the desired mechanical and microstructural properties of sinters, and inversely, is important in the context of industrial practice as well as in laboratory work. Artificial intelligence, specifically artificial neural networks, have proven their effectiveness in this task. The criteria for assessing the effectiveness of ANNs were the coefficient of determination (R^2^) and the sum of squares error (SSE), obtained at the level of R^2^ > 0.95 and SSE < 0.02, for the best ANN collectively predicting output values.

Separating the ANN effectiveness into individual outputs showed significant results for (1) the surface-affected zone (R^2^ > 0.999), followed by (2) density (R^2^ > 0.9988), (3) open porosity (R^2^ > 0.999), (4) porosity (R^2^ > 0.995), and (5) hardness (R^2^ > 0.9735). Therefore, the prediction of microstructural properties has key potential, and the prediction of mechanical properties does as well. Slightly worse results in hardness predictions may be due to the specific nature of the measurements taken during data collection for subsequent neural network training. Density measurements are more accurate and repeatable compared to hardness, which depends on the microstructure at the measurement point. Even with multiple hardness measurements at different points, the average of several points on a single surface is still considered rather than the overall surface area.

The influence of sintering parameters such as temperature and holding time on material properties such as the density, porosity and surface-affected zone depth is well established. Consequently, the predictive effectiveness of the network for these parameters is very high with an R^2^ value of approximately 0.999. As these values increase, the density and surface-affected zone increase and the porosity decreases. In the case of hardness, however, the relationship is more complex. An increase in sintering temperature and holding time initially results in increased hardness due to the superior material densification and lower porosity in the microstructure. However, this only occurs up to a certain point; thereafter, grain growth becomes the dominant factor, lowering hardness. This complex relationship can be observed when sintering at 1150 °C, where hardness initially increases due to increasing density but then decreases as a result of grain growth. The complex influence of sintering parameters on material hardness results in lower prediction accuracy compared to that of other properties analyzed in this paper. As a result, the overall performance of the model is slightly lower with an R^2^ value of approximately 0.9735. The observed standard deviations in hardness measurements can propagate through the ANN and affect prediction accuracy, which can partly explain the lower R^2^ value for hardness.

The effectiveness of ANNs in optimizing SPS parameters is high; for both sintering temperature and holding time, it is R^2^ > 0.95. Therefore, it can be concluded that the sintering temperature and holding time are strongly related to the mechanical and microstructural properties of the sintered metal powders. This also provides reliable evidence for the possibility of indicating SPS parameters depending on the expected parameters of the sinters.

Leave-one-out cross-validation (LOOCV) provided a robust evaluation strategy for the testing dataset. This approach ensured that each sample was tested exactly once on a model trained without that sample, allowing errors to be calculated exclusively on unseen data and thus reflecting the models’ true predictive outcomes. The results also highlight the sensitivity of neural networks to output scale variability, indicating that additional data or further model regularization could improve the models’ performance. Two different MLP architectures were evaluated. The first model, with two input neurons and five output neurons, was designed to predict sintering properties including density, open porosity (measured via the Archimedes method), porosity from image analysis, Vickers hardness, and the surface-affected zone. For this model, the mean absolute error (MAE) per output ranged from 0.30 for density to 34.93 for the surface-affected zone with corresponding root mean square errors (RMSEs) between 0.53 and 46.90. The global MAE and RMSE values, averaged across all five outputs, were 11.49 and 14.82, respectively. The second model, reversing the predictive role of inputs and outputs, used five inputs to predict the sintering temperature and sintering time. This configuration yielded MAE values of 22.81 for temperature and 3.05 for time with RMSE values of 34.26 and 4.89, resulting in global MAE and RMSE values of 12.93 and 19.57, respectively. Overall, the LOOCV framework provided a comprehensive evaluation, demonstrating that both models delivered quantitatively meaningful predictions on samples not used during training. The first model, taking two inputs (SPS parameters) to predict five sintering properties, demonstrated high accuracy for density and porosity measures, while larger deviations were observed for hardness and the surface-affected zone, as reflected in the MAE and RMSE values. These discrepancies among individual outputs can be attributed to the differing numerical ranges of the properties. The outputs with larger absolute values produced higher errors. The global error metrics indicate that on average, the model captured the main trends across all outputs in a reasonable manner. The second model, with five inputs (sintering properties) and two outputs predicting sintering temperature and time, showed that the predictions of sintering time were more accurate than those of temperature. However, the model maintained acceptable predictive quality. Together, these results illustrate that both models provide reliable estimates on samples not seen during training, highlighting their usefulness in evaluating material processing parameters and resulting properties. These errors (MAE and RMSE) are practically acceptable, as the models serve as a tool to support the design of sintering processes and the resulting material properties, highlighting trends.

The predictive effectiveness of the artificial neural networks was assessed based on two fundamental criteria: the sum of squared errors (SSE) and the coefficient of determination (R^2^) between the experimental and predicted results. Based on the evaluation criteria, the most effective network architectures and parameter configurations were identified:

- Types of ANNs—the better performance of the MLP type compared to the RBF results from its ability to capture global nonlinear relationships between SPS parameters and the microstructural and mechanical properties of the material. In contrast, RBF networks mainly model relationships locally and often require an expanded dataset to achieve comparable predictive accuracy. The MLP network provides greater architectural flexibility and the ability to capture global data patterns, whereas RBF networks exhibit faster training and higher local sensitivity, which is more applicable to tasks such as anomaly detection. The first four networks are MLPs, while the RBF network ranked fifth. Its predictive performance is lower with an R^2^ approximately 8% below that of the MLP network;
- ANN learning algorithms—the Broyden–Fletcher–Goldfarb–Shanno (BFGS) algorithm provides the best performance. This can be attributed to its quasi-Newton optimization approach, which efficiently approximates second-order derivatives, allowing faster and more stable convergence compared to first-order methods such as the scaled conjugate gradient. The BFGS algorithm is also less sensitive to the choice of learning rate and tends to perform better on moderately sized datasets, and it is particularly suitable for SPS process modeling;
- Number of hidden neurons—networks with four to seven hidden neurons achieve better predictive performance than those with only one to three neurons. Fewer neurons limit the network’s capacity to capture nonlinear relationships between SPS parameters and material properties, potentially causing underfitting. In contrast, four to seven neurons offer enough capacity to model these dependencies. Consequently, the number of hidden neurons was not increased beyond this range (4–7) to avoid unnecessary complexity and the risk of overfitting;
- Activation functions for hidden neurons—hyperbolic tangent, exponential, and sigmoid functions perform better than sine, linear, or Gaussian functions. The better performance of these functions stems from their continuous, nonlinear properties, which provide stable gradients and enable the network to accurately capture complex relationships between SPS parameters and sintering properties. In contrast, the linear function cannot model nonlinearities, while sine and Gaussian functions may cause oscillations or local sensitivity;
- Activation functions for output neurons—sigmoid, linear, and hyperbolic tangent functions perform better for stable predictions across the data range. Linear activation generates outputs without range constraints, whereas sigmoid and tanh functions are appropriate for normalized data. In contrast, sine and Gaussian functions exhibit local or oscillatory behavior, leading to unstable predictions and a weaker modeling of global relationships. Although linear activation does not introduce nonlinearity and limits the capacity of hidden layers, it remains advantageous in the output layer for regression tasks.

In summary, the most appropriate ANN configuration consists of an MLP architecture with the BFGS training algorithm, four to seven hidden neurons, hyperbolic tangent, exponential, or sigmoid activation functions for the hidden layer, and sigmoid, hyperbolic tangent, or linear activation functions for the output layer.

Linear regression plots show the convergence between the real and predictive results. Values closer to the trend line indicate a very good fit, and the coefficient of determination is close to 1. The best ANN determined by R^2^ and SSE is characterized by a very strong fit between the predicted results and the real results, and it is only slightly worse for hardness prediction. The discrepancies around the trend line are noticeable, becoming larger with each successive ANN model proposed. The distribution of points along the trend line is even and symmetrical, and no overestimation or underestimation is observed.

While genetic algorithms (GA) and particle swarm optimization (PSO) are widely used optimization methods, their direct application to the prediction of SPS sintering properties presents several limitations. GA and PSO are optimization techniques rather than predictive models, and their application would require coupling with an additional predictive model to estimate material properties, whereas the ANNs directly capture the nonlinear relationships between SPS parameters and sinter characteristics (mechanical and microstructural properties) and provide predictive results. Additionally, the interpretability of GA and PSO results is limited, as these methods provide optimal parameter sets without readily revealing the influence of individual SPS parameters on the predicted outcomes. Considering these factors, the use of ANNs in this work provides a more direct and practical approach for predicting material properties after SPS processes.

Artificial neural networks can support the design of spark plasma sintering processes as well as the quality control of sintered powders, supporting long-term and demanding mechanical and material tests of metallographic specimens. Artificial intelligence methods meet the challenges of modern industry.

## 5. Conclusions

The study contributes to measurement science and practice by presenting a novel approach for the intelligent modeling and optimization of the mechanical and microstructural properties of sinters produced by spark plasma sintering from 316L stainless steel powder. The relationships between SPS parameters and the mechanical and microstructural properties of sintered powders can be nonlinear, complex, and influenced by multiple factors. In this context, artificial intelligence methods offer a promising alternative to classical mathematical modeling. The reliability of predictions depends on the ANN model but primarily on the empirical, reliable, and repeatable dataset used. In these studies, the experimental dataset contributed to the development of effective neural networks. It is worth emphasizing that the developed ANNs can both predict the properties of sintered powders based on SPS parameters and indicate the SPS parameters required to achieve the desired properties of the sinters. ANNs can be expanded with additional input and output data and adapted to evaluate other materials. The mechanical and microstructural properties of engineering materials determine the functional performance of their surfaces, influencing wear resistance. These studies are innovative in several respects. Here, a method is described for predicting both the SPS process parameters and the properties of sintered powders, taking into account the comprehensive experimental results of 316L steel, which had not been previously modeled by artificial intelligence. Of particular note is surface-affected zone depth, which has not been assessed using neural networks in publications but is important in surface phenomena such as corrosion resistance.

The detailed conclusions from these studies are as follows:

- Artificial neural networks enhance experimental-based frameworks for modeling and optimizing the spark plasma sintering process and sintered metal powders.
- The developed measurement system identifies the relationships between the sintering temperature, holding time, and material properties (density, porosity, hardness, and surface-affected zone depth), providing reliable predictive values.
- The developed models are valid within industrial measurement systems, and they are limited to the tested SPS setup and the experimentally investigated process parameter window.
- Linear regression analysis confirmed convergence between the real (experimental) measurements and model predictions.
- The prediction of the mechanical and microstructural properties of sintered powders based on spark plasma sintering parameters is effective (R^2^ > 0.95 and SSE < 0.02).
- The most effective network for predicting sintered powder properties is the MLP 2-5-5 with a hidden layer activated by the hyperbolic tangent function and an output layer activated by the sigmoid function.
- The recommendation of SPS parameters based on the expected sintering properties is strong (R^2^ > 0.95 and SSE < 0.02).
- The best network for indicating spark plasma sintering parameters is the MLP 5-4-2 with a hidden layer activated by the exponential function and an output layer activated by the linear function.
- Leave-one-out cross-validation (LOOCV) on limited data (14 observations) showed that the first MLP model predicted sintering properties with a global MAE of 11.49 and RMSE of 14.82, while the second model predicted a sintering temperature and time with a global MAE of 12.93 and RMSE of 19.57; higher errors occurred for outputs with larger numerical ranges, but the overall trends were well captured.
- Increasing the sintering temperature and holding time is directly proportional to the density and hardness of the sinters and inversely proportional to the porosity.
- Prediction of the possible carbide precipitation in the near-surface zone is important for functional aspects such as resistance to corrosion.

## Figures and Tables

**Table 1:** Chemical composition of 316L SS powder.

Element	Fe	Cr	Ni	Mo	Mn	Si	N	O	P	C	S
Mass %	Balance	16.00–18.00	10.00–14.00	2.00–3.00	≤2.00	≤1.00	≤0.10	≤0.10	≤0.05	≤0.03	≤0.03

**Table 2:** Activation functions [59].

Activation Function	Mathematical Expression	Output Values Range
Linear	$f\left(x\right)=x$	–∞, +∞
Sigmoid	$f\left(x\right)=\frac{1}{1-e^{-1}}$	0, 1
Exponential	$f\left(x\right)=e^{-x}$	0, +∞
Hyperbolic tangent	$f\left(x\right)=\frac{e^{x}-e^{-x}}{e^{x}+e^{-x}}$	–1, 1
Sine	$f\left(x\right)=sin(x)$	–1, 1
Gaussian	$f\left(x\right)=exp\left(-\frac{{\left(x-c\right)}^{2}}{2\sigma^{2}}\right)$	0, 1

**Table 3:** Results of measurements of sintering properties in relation to SPS parameters.

SPS Process Parameters		Properties of the Material				
Sintering Temperature	Holding Time	Density	Open Porosity (Archimedes Method)	Porosity (Image-Analysis Area Fraction)	Vickers Hardness	Surface-Affected Zone
°C	min	g/cm^3^	%	%	HV_0.3_	µm
950	15	7.27 ± 0.04	7.79 ± 0.07	5.55 ± 0.33	174 ± 5	155.51 ± 12.1
	5	7.01 ± 0.04	11.08 ± 0.07	6.65 ± 0.41	114 ± 8	76.16 ± 4.42
	1	6.51 ± 0.04	17.38 ± 0.07	12.52 ± 0.5	101 ± 4	58.57 ± 6.51
1000	15	7.65 ± 0.04	2.93 ± 0.07	2.47 ± 0.32	216 ± 12	213.43 ± 5.89
	5	7.40 ± 0.04	6.09 ± 0.07	4.06 ± 0.08	156 ± 9	120.27 ± 3.9
	1	6.90 ± 0.04	12.48 ± 0.07	9.27 ± 0.48	126 ± 7	76.12 ± 3.21
1100	15	7.88 ± 0.04	0.03 ± 0.07	0.38 ± 0.02	182 ± 3	564.35 ± 22.87
	5	7.77 ± 0.04	1.37 ± 0.07	1.23 ± 0.04	196 ± 3	298.06 ± 9.73
	1	7.62 ± 0.04	3.24 ± 0.07	1.99 ± 0.07	167 ± 5	177.03 ± 6.84
1150	15	7.85 ± 0.04	0.42 ± 0.07	0.29 ± 0.09	225 ± 4	676.03 ± 33
	5	7.85 ± 0.04	0.42 ± 0.07	0.67 ± 0.19	205 ± 4	387.31 ± 19.58
	1	7.81 ± 0.04	0.92 ± 0.07	0.82 ± 0.12	180 ± 2	276.79 ± 11.93
975	12	7.37 ± 0.04	6.45 ± 0.07	4.94 ± 0.34	185 ± 5	144.85 ± 6.02
1075	3	7.44 ± 0.04	5.60 ± 0.07	3.77 ± 0.72	150 ± 4	144.8 ± 8.9

**Table 4:** The best networks for predicting the relationship between SPS parameters (inputs) and sinters’ properties (outputs).

Number of ANN	Architecture	R^2^	SSE	Training Algorithm	Activation Function in the Hidden Layer	Activation Function in the Output Layer
1	MLP 2-5-5	0.9967	0.0015	BFGS	Hyperbolic tangent	Sigmoid
2	MLP 2-7-5	0.9943	0.0025	BFGS	Exponential	Linear
3	MLP 2-5-5	0.9873	0.0058	BFGS	Sigmoid	Hyperbolic tangent
4	MLP 2-7-5	0.9843	0.0069	BFGS	Sigmoid	Sigmoid
5	MLP 2-6-5	0.9791	0.0092	BFGS	Sigmoid	Hyperbolic tangent

**Table 5:** Real versus predicted properties of sintered powders.

Density		Open Porosity (Archimedes Method)		Porosity (Image-Analysis Area Fraction)		Vickers Hardness		Surface-Affected Zone	
g/cm^3^		%		%		HV_0.3_		µm	
Real	Predicted	Real	Predicted	Real	Predicted	Real	Predicted	Real	Predicted
7.266	7.275	7.794	7.682	5.546	5.712	174.4	177.151	155.510	128.339
7.007	7.008	11.076	11.074	6.655	6.653	114.2	114.062	76.160	58.794
6.510	6.510	17.381	17.381	12.522	12.522	101.4	101.400	58.569	58.569
7.649	7.643	2.930	3.009	2.467	2.460	215.6	214.726	213.431	220.464
7.400	7.398	6.089	6.120	4.061	4.178	156.0	156.391	120.274	139.182
6.896	6.891	12.484	12.550	9.268	9.144	125.8	124.501	76.124	86.555
7.878	7.866	0.026	0.177	0.381	0.368	181.8	191.571	564.354	563.359
7.772	7.787	1.375	1.184	1.227	0.892	196.2	190.317	298.060	285.577
7.625	7.618	3.237	2.696	1.993	1.895	166.8	159.853	177.028	193.445
7.847	7.878	0.423	0.026	0.293	0.293	225.0	223.616	676.029	676.029
7.847	7.831	0.422	0.615	0.668	0.592	204.6	190.744	387.314	390.206
7.807	7.787	0.924	1.184	0.822	0.892	179.8	190.317	276.785	285.577
7.372	7.370	6.451	6.467	4.941	4.761	185.4	183.094	144.848	142.501
7.438	7.446	5.604	5.511	3.769	3.841	150.2	150.092	144.804	138.960
SSE = 0.00225		SSE = 0.647432		SSE = 0.226932		SSE = 498.38		SSE = 2210.46	
MSE = 0.000161		MSE = 0.046245		MSE = 0.01621		MSE = 35.60		MSE = 157.89	
RMSE = 0.0127		RMSE = 0.215		RMSE = 0.1273		RMSE = 5.97		RMSE = 12.57	

**Table 6:** The best network models for predicting the relationship between sintered powders’ properties (input) and SPS parameters (output).

Number of ANN	Architecture	R^2^	SSE	Training Algorithm	Activation Function in the Hidden Layer	Activation Function in the Output Layer
1	MLP 5-4-2	0.999645	0.000111	BFGS	Exponential	Linear
2	MLP 5-7-2	0.996893	0.000980	BFGS	Hyperbolic Tangent	Sigmoid
3	MLP 5-4-2	0.994557	0.001777	BFGS	Sigmoid	Exponential
4	MLP 5-7-2	0.987252	0.004808	BFGS	Exponential	Sigmoid
5	RBF 5-7-2	0.973816	0.008027	RBFT	Gaussian	Linear

**Table 7:** Real versus predicted sintering parameters.

Sintering Temperature			Sintering Time		
°C			min		
Real	Predicted	Error (Difference)	Real	Predicted	Error (Difference)
950	953.5	0.37%	15	14.9	0.67%
950	949.4	0.11%	5	5.0	0.00%
950	947.7	0.24%	1	1.0	0.00%
1000	1001.0	0.10%	15	14.9	0.67%
1000	1002.9	0.29%	5	4.9	2.00%
1000	1001.3	0.13%	1	0.9	10.00%
1100	1100.0	0.00%	15	15.0	0.00%
1100	1095.4	0.49%	5	5.3	6.00%
1100	1092.8	0.66%	1	1.0	0.00%
1150	1149.9	0.01%	15	15.0	0.00%
1150	1152.2	0.19%	5	4.8	4.00%
1150	1150.2	0.02%	1	1.0	0.00%
975	971.6	0.36%	12	12.2	1.67%
1075	1074.7	0.02%	3	3.0	0.00%
SSE = 118.54			SSE = 0.21		
MSE = 8.47			MSE = 0.015		
RMSE = 2.91			RMSE = 0.122		

**Table 8:** Leave-one-out cross-validation (LOOCV) results for predicting sintering properties.

	Density	Open Porosity (Archimedes Method)	Porosity (Image-Analysis Area Fraction)	Vickers Hardness	Surface-Affected Zone
**MAE per output**	0.2989	1.6528	1.0551	19.5102	34.9332
**RMSE per output**	0.5273	2.5178	1.2875	22.8851	46.8965
**Global MAE** **(averaged across outputs)**	11.4900				
**Global RMSE** **(averaged across outputs)**	14.8228				

**Table 9:** Leave-one-out cross-validation (LOOCV) results for predicting spark plasma sintering properties.

	Sintering Temperature	Sintering Time
**MAE per output**	22.8134	3.0516
**RMSE per output**	34.2605	4.8886
**Global MAE** **(averaged across outputs)**	12.9325	
**Global RMSE** **(averaged across outputs)**	19.5746	

## Data Availability

The original contributions presented in the study are included in the article; further inquiries can be directed to the corresponding author.
